
JOURNAL INFORMATION
==============================
NLM Title Abbreviation: Med Exam (Phila)
NLM Journal ID: 0017413

Medical Examiner (Philadelphia, Pa.)

Publisher: Biomedical Journal Digitization Project

ARTICLE INFORMATION
==============================
PMCID: PMC10285833
PMID: 38120184
Article version: 1
Subjects: Record of Medical Science

Successor to Breschet

Print publication date: 1846 May
Volume: 2
Volume Series: New Ser
Issue: 17
First page: 332
Last page: 332
License: This work is free of known copyright restrictions. For more information, please see PMC Back Issue Digitization.
License URL: https://creativecommons.org/publicdomain/mark/1.0/

==============================

---

*Premature Interments.*—It is stated that the cases of premature interment in France, prevented by fortuitous circumstances, amount, since the year 1833, to 94. Of these, 35 persons awoke of themselves from their lethargy at the moment the funeral ceremony was about to commence; 13 recovered in conseqnence of the affectionate care of their families; 7 in consequence of the fall of the coffins in which they were inclosed; 9 owed their recovery to wounds inflicted by the needle in sewing their winding sheet; 5 to the sensation of suffocation they experienced in their coffin; 19 to their interment having been delayed by fortuitous circumstances; and 6 to their interment having been delayed in consequence of doubts having been entertained of their death.—*Prov Med. and Surg. Journ.*

---

*Royal College of Surgeons.*—Mr. J. H. Green has been elected a member of the Court of Examiners of the Royal College of Surgeons, to fill up the vacancy occasioned by the resignation of Sir Benjamin Brodie.—*London Med. Gaz.*

---

*Successor to Breschet.*—The concours for the Chair of Anatomy in the Faculty of Medicine, rendered vacant by the death of M. Breschet, has terminated in the election of M. Denonvilliers by a majority of nine out of twelve votes. M. Chassaignac and Gosselin are reported to have highly distinguished themselves in this concours.—*Ibid.*

---

**ELLIS'S MEDICAL FORMULARY.**

CORRECTION.—The Publishers of this Work respectfully request those persons who have the seventh edition, to correct a typographical error for the "MEDICATED HYDROCYANATE OF POTASSA," at page 83; wherein the symbol for an ounce is used in place of that for a drachm. The following is the correct prescription, and corresponds with the proportions directed in all the previous editions of the Work:

℞. Potass. hydrocyan. medicati, ʒj.
Aquæ destillatæ, Oj.
Sacchari purificati, ℥iss.
Fiat solutio.—Dose, a table-spoonful, night and morning.
